# Passport Officers’ Errors in Face Matching

**DOI:** 10.1371/journal.pone.0103510

**Published:** 2014-08-18

**Authors:** David White, Richard I. Kemp, Rob Jenkins, Michael Matheson, A. Mike Burton

**Affiliations:** 1 School of Psychology, The University of New South Wales, Sydney, Australia; 2 Department of Psychology, University of York, York, United Kingdom; 3 The Australian Passport Office, Department of Foreign Affairs and Trade, Barton, Australia; 4 School of Psychology, University of Aberdeen, Aberdeen, United Kingdom; University of Lincoln, United Kingdom

## Abstract

Photo-ID is widely used in security settings, despite research showing that viewers find it very difficult to match unfamiliar faces. Here we test participants with specialist experience and training in the task: passport-issuing officers. First, we ask officers to compare photos to live ID-card bearers, and observe high error rates, including 14% false acceptance of ‘fraudulent’ photos. Second, we compare passport officers with a set of student participants, and find equally poor levels of accuracy in both groups. Finally, we observe that passport officers show no performance advantage over the general population on a standardised face-matching task. Across all tasks, we observe very large individual differences: while average performance of passport staff was poor, some officers performed very accurately – though this was not related to length of experience or training. We propose that improvements in security could be made by emphasising personnel selection.

## Introduction

In modern society, our security relies on accurate identification. Whenever we cross a border, apply for a passport or access secure premises, we are required to prove our identity. Although there is increasing interest in different biometric markers to support this process, the most prevalent means of identification is verification of photo-ID, and we rely on trained specialists to perform this task accurately. However, experiments consistently show that viewers are poor at matching photos of unfamiliar faces [1]–[6], making surprisingly large numbers of errors even when high quality photos, taken on the same day, are presented side-by-side. Moreover, matching a live person to a photo is no easier [4]–[6], a result which brings the use of photo-ID into question.

Experimenters have typically measured face matching performance in non-specialist, student volunteers. It is critical to know whether people with specialist training and experience can perform the task well, and in particular, whether they perform better than standard experimental groups – about whom there is now a large body of evidence. To address this, we examined the ability of passport-issuing government employees to match faces – using standard laboratory tasks, and genuine government approved photo-ID.

We expected that accuracy of these passport officers would exceed that of student participants in laboratory settings. There are two reasons for this expectation. First, experience performing unfamiliar face matching tasks as part of daily work might improve accuracy. It is well known that people are extremely accurate at matching *familiar* faces [7], making their poor performance with unfamiliar faces all the more striking [8]. Perhaps one factor contributing to the difficulty of *unfamiliar* face matching is that this task is rarely encountered by people in their daily experience: the vast majority of face processing is directed towards faces that we know [9]. Experimental participants are often surprised by the difficulty of unfamiliar face matching [1], suggesting that poor performance in laboratory tests may stem, in part, from the novelty of the tasks. This novelty is lost in occupational settings.

Second, the passport staff we tested had all received training in facial image comparison as part of their employment. The purpose of this training is to equip passport officers with more effective strategies for comparing facial images. Reports of effective training for unfamiliar face matching tasks are rare, and some null results have been reported [10], [11]. However, we have shown in recent work that face matching performance can be improved by some types of training [12]. Here we asked whether occupational training enhances performance in this task.

## Participants

The studies reported here took place during normal working hours at Sydney Passport Office. Time for testing was generously donated by the participants and their employer, the Department of Foreign Affairs and Trade. We tested passport officers’ ability to make same/different identity judgments to either person-photo pairs (*Person-to-Photo test*), or photo-photo pairs (*Photo-to-Photo test, Glasgow Face Matching Test*). Participants were 49 passport officers (32 Female, Mean age = 46.8, SD = 11.3) whose main responsibility is to assess the eligibility of passport applicants. All participants routinely confirm identity by checking people against their ID photos (when citizens apply for passports in person), and make photo-to-photo comparisons – in the case of passport renewals, and when checking for potentially fraudulent duplicate applications.

**Figure 1:**
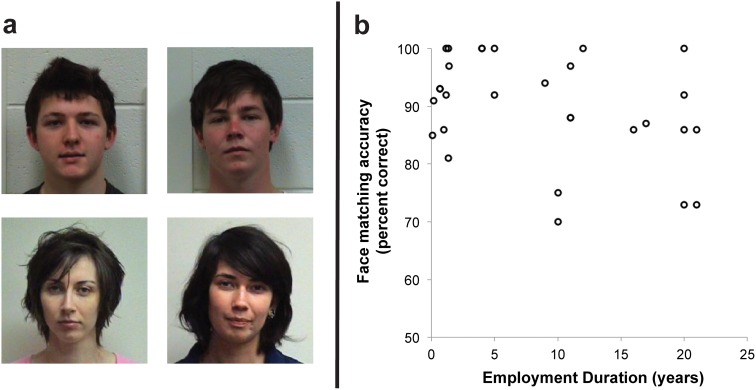
Example photo-ID and results for *Photo-to-Person* test. (a) Example valid ID-photos (left column) alongside invalid photos of foil identities (right column). (b) Performance on *Person-to-Photo test* as a function of Employment Duration (note three participants were excluded from this analysis because the duration of their employment was unknown). The individuals pictured in this figure have given written informed consent (as outlined in PLOS consent form) for their images to be published.

Participants had considerable experience in this role (mean = 8 years and 7 months), though there were large differences within the group, ranging from employees with over twenty years experience, to relatively recent recruits (sd = 7 years 7 months; see Fig. 1b).

All but three passport officers had completed a short training module on identity verification from photographs as part of their employment. This training encouraged a feature-by-feature approach to facial image comparison. For example, it instructed staff to “break the face into parts and compare each segment”, and to avoid fixating on the “triangle of recognition” (defined as the area triangulated by the eyes and the mouth). A number of other agencies provide similar training to their staff. Removing the three new recruits that had not completed this training did not change the outcome of any analyses reported in this paper.

### Ethics Statement

This study was approved by the Human Research Ethics Committee at the University of New South Wales. All participants provided written informed consent and appropriate photographic release (as outlined in PLOS consent form).

## Person-to-Photo Test

### Participants and stimuli

Thirty passport officers took part in this test (21 Female, Mean age = 48.0, SD = 11.7). In addition, we recruited 34 students (17 females) to act as ID-card bearers (henceforth ‘applicants’) for the live identification task. For each of these people, we took an ID image from a short video clip that was recorded on a high quality digital video camcorder. We then extracted from this video sequence a frame showing full-face pose and neutral expression, in accordance with international photo-ID guidelines (examples are shown in Fig. 1a). All images were cropped in square aspect ratio and scaled to 200 by 200 pixels for presentation on a computer monitor. For each applicant, the experimenter chose a foil (i.e. fraudulent) image by picking the student whose appearance was most subjectively similar to the applicant’s ID photo.

In some regards, methods of stimulus preparation made this task much *easier* than many real-world identification tasks. First, all photos of applicants were taken just a few days prior to the experiment. In real life, photo-ID may be several years old. Second, selection of foils was severely restricted. Real world users of false photo-ID have an interest in targeting people who resemble them, or adjusting their own appearance to match the false ID. In this study, foils were chosen from a very small sample of 17 people of the same sex as the applicant, and the group was very diverse, being an arbitrary sample of students (Fig. 1a).

### Design and Procedure

All testing took place in the workplace (Sydney Passport Office) on a normal working day. Participants were tested six at a time, and sat behind their own desks with laptops that were clearly marked with a number from 1 to 6. Applicants were issued with cards showing a five-digit code number. They were given written instructions specifying the order in which they should approach each desk, and which card should be presented. The passport officer took the presented card, and entered its code number onto a laptop, which then displayed a photo, either valid or invalid. The applicants could not see the laptop screen, and did not know on any particular trial whether they were presenting genuine or fraudulent ID. This ensured that measures of face matching accuracy were not confounded by cues to identity from card bearer’s behavior.

On each trial, passport officers could view the photo for ten seconds, after which it disappeared. They were invited to indicate whether this was a valid or invalid card, within the viewing period. Across the experiment, all applicants presented valid and invalid ID equally often. The order in which applicants visited the desks was different for each of six test sessions. Because testing took place on a normal working day, experimental sessions were time-limited. This meant that it was not possible for all passport officers to see all applicants, and differences in work rate led to some variability in the number of trials completed. Across the experiment, participants completed on average 13.2 matching trials (SD = 4.12) and 13.5 mismatching trials (SD = 3.22).

### Results

Overall, passport officers (n = 30) made an average of 10% errors on the *Person-to-Photo test*. 6% of valid photos were wrongly rejected, and 14% of fraudulent photos were wrongly accepted. Given the constrained selection of imposters in this study, it is perhaps surprising that trained staff accepted fraudulent ID so frequently. Because of the range in duration of passport officers’ professional experience, we next tested whether experience predicted performance on the task. There was no relationship between employment duration and face matching accuracy [n = 27, *Spearman’s rho* = −0.242, *p*>0.05] (Fig. 1b). Thus, performance on this task does not appear to be determined by either experience or by current training methods.

## Photo-to-Photo Test

Some time after the initial *Person-to-Photo* test, we returned to the same workplace and set passport officers a *Photo-to-Photo test*. Photo-ID typically remains valid for several years. For this reason, personnel attempting to verify ID have to deal with a wide range of images, and often do not know when a particular photo was taken. Age-related changes in appearance are known to have a large effect on matching accuracy [13]. Here we examined performance across a relatively short, known interval of two years.

We were unable to test a control group as part of the *Person-to-Photo* test because ‘applicants’ in this study were not able to return for a second test. Therefore, in the *Photo-to-Photo* test, we also made a direct comparison between passport officers and a group of non-specialist student participants, representing by far the most commonly sampled population for psychological experiments.

### Participants

Twenty-seven passport officers took part in this test, from the same population as the previous study (22 Female, Mean age = 45.5, SD = 10.9; see above). Of these, 10 had participated in the *Person-to-Photo* test, two years earlier. Student participants were 38 volunteer students from the University of New South Wales (26 Female; Mean Age = 18.9, SD = 1.3).

### Stimuli

**Figure 2:**
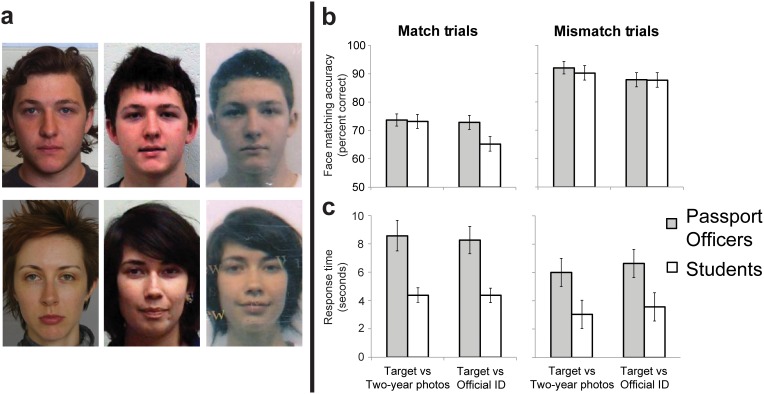
Example image pairs and results for *Photo-to-Photo* test. (a) Representative match pairs (top row) and mismatch pairs (bottom row) from experimental conditions. Targets (left column) were new photos, and these were matched against two-year-old photos (middle column) or official ID photos (right column). (b) Mean accuracy and (c) response time data for passport officers and students in the *Photo-to-Photo test*. Error bars represent SEM.

Stimuli were photographs of the student applicants from the *Photo-to-Person* test. All those who had taken part in the study were contacted again two years later, and asked if they would be willing to supply two further images of their face for use in a matching experiment. They were asked to supply (i) a photo scanned from current official ID (driving license or passport), and (ii) a new image taken using a camera-phone or web-cam, taken under good lighting conditions, with a neutral expression and looking straight at the camera. Of the 34 applicants that took part in the earlier test, 21 supplied images for use in the *Photo-to-Photo test*. To create this test, we used the new photographs as targets (Fig. 2a, left), for participants to compare with old experimental photographs (taken two years previously, Fig. 2a, middle) and official photo-ID (taken an indeterminate time previously, but currently valid, Fig. 2a, right). For mismatch trials, we paired target images with the corresponding images of foil identities. As previously, these were chosen to appear most similar to the target from within the group of 34 identities – a severely restricted set.

### Design and procedure

All participants completed a computer-based face matching task. As with previous experiments, testing took place in the workplace on a normal working day. On each trial, a target image appeared on the left of the computer screen, with a comparison image from one of two stimulus conditions (two-year-old photo or official ID photo) on the right (Fig. 2a). Participants had to decide if the images depicted the same person or two different people. The task was self-paced, and we encouraged participants to respond accurately. Subjects viewed all combinations of pairs (match/mismatch, two-year-old photo/official-ID), giving a total of 84 trials presented intermixed in a random order. This resulted in a 2×2×2 mixed design, with Participant Group (passport officers vs students) as the between-subjects factor.

### Results

Percentage accuracy rates were analyzed using 2×2×2 mixed ANOVA, with factors Trial Type (match/mismatch), Photo Type (Two-year old/Official ID) and Participant Group (passport officers/students; see Fig. 2b). This analysis relevealed a non-significant main effect of group [F (1,63) = 2.35; p>0.05; η^2^ = 0.037], however main effects were qualified by a significant three-way interaction between factors [F (1,63) = 5.66; p<0.05; η^2^ = 0.089].

To explore the three-way interaction, we analyzed accuracy separately for match and mismatch trials with 2×2 mixed ANOVA.

For match trials, overall performance was poor (70.9%). The main effect of Participant Group was non-significant [F (1,63) = 1.25, p>0.05, η^2^ = 0.019], due to overall accuracy on match trials being similar for Passport Officers and students. However, the main effect of Photo Type was significant [F (1,63) = 10.4, p<0.05, η^2^ = 0.165], with photo-ID images matched less accurately than photos taken in our laboratory. There was also a significant interaction between Participant Group and Photo Type [F (1, 63) = 6.64, p<0.05, η^2^ = 0.105], due to the student group being particularly poor at matching the new photos to official ID (simple main effect of Photo Type for students, F(1,63) = 36.5, p<0.05, η^2^ = 0.321, for passport officers, F<1).

For mismatch trials, overall performance was higher (89.4%), reflecting an overall tendency to perceive the photos as showing different people. This result might be explained by the fact that photo-identification documents become less representative of the card holder over time, but do not become more like foil identities (see also [13]). There was a main effect of Photo Type, whereby matches against official ID were less accurate than matches against two-year old photos [F (1,63) = 11.2, p<0.05, η^2^ = 0.177]. There was no main effect of Participant Group and no interaction (Fs<1). As in the previous study, we found that employment duration for the passport officer group did not predict overall accuracy on the task [*Spearman’s rho*<0.001, p>0.05].

In this experiment, we were primarily interested in response accuracy. However, we also analyzed participants’ response times (Fig. 2c), to test whether passport officers devoted more time to making face matching decisions than student controls. The test was self-paced, and because we expected that passport officers would have greater motivation to perform well, it was important to check for differences in decision time. The main effect of Participant Group was significant [F (1,63) = 24.1, p<0.05, η^2^ = 0.381] with passport officers taking much longer to make decisions than students. The main effect of Trial Type was also significant, with participants taking longer for match trials than for mismatch trials [F (1,63) = 17.0, p<0.05, η^2^ = 0.270]. There were no significant interactions between Participant Group and any other factor. Thus, passport officers took significantly longer than students to make their decisions (Fig. 2a), and this cost was paired with a small accuracy advantage in one of the four conditions of the *Photo-to-photo* test (Fig. 2b).

Even when using official photo-ID, which had already been approved by government agencies, experienced operators made a large number of errors, as did our non-specialist group.

## Glasgow Face Matching Test

We also measured passport officers’ accuracy on a standard psychometric test of face matching ability, the *Glasgow Face Matching Test* (GFMT [3]), to compare passport officers’ performance against established population norms.

### Method

Thirty passport officers completed the short version of the GFMT (20 Female, Mean age = 47.4, SD = 11.9). The GMFT was administered immediately prior to the debrief session in the *Person-to-Photo test* (which took place one week after the *Person-to-Photo test* session). Two participants were replaced because they were absent from work on this day. In the short version of the GFMT, participants view 40 pairs of faces, half of which are same-person pairs and half of which are different-person pairs. The photos were taken a few minutes apart, but with different cameras, which makes the match non-trivial (for details see [3]). Photo pairs from the GFMT were projected onto a large viewing screen for 6 seconds each. For each pair, participants indicated whether the pair of faces belonged to the same or different people. All thirty passport officers were tested together, but made their responses individually, with no conferring.

### Results

**Figure 3:**
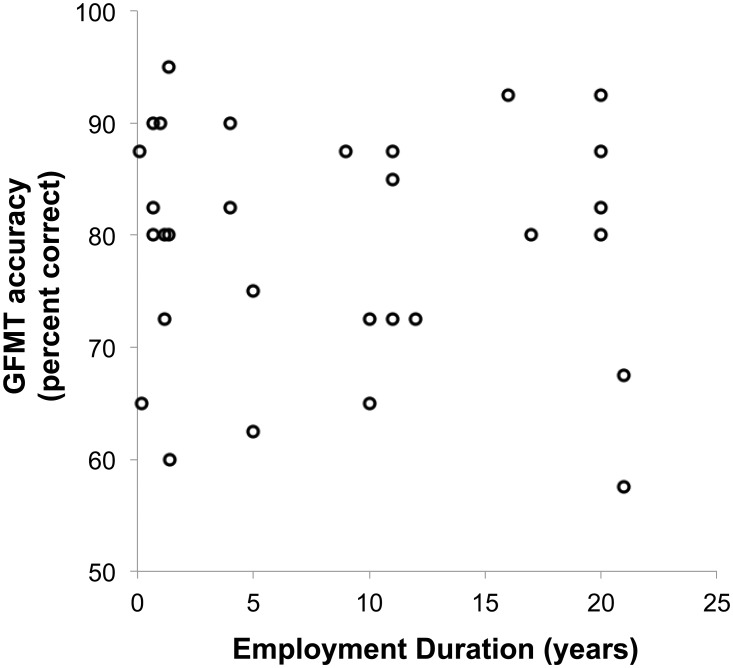
Performance on the GFMT as a function of Employment Duration.

Overall, GFMT performance in passport officers (M = 79.2%, SD = 10.4%) did not differ significantly from normative scores (M = 81.3%, SD = 10.4%, n = 194; [3]), [t (222) = −1.097, p>0.05]. Although this is very surprising, it is completely consistent with data from the other tests. Again, there was no relationship between experience and accuracy (n = 30, *Spearman’s rho* = −0.105, *p*>0.05; Fig. 3).

Because 28 passport officers that completed the GFMT had also completed the photo-to-person test, we were able to examine correlations between these two tasks. Performance on the GFMT predicted performance in the photo-to-person matching task, but only for mismatch trials (n = 28, *Spearman’s rho* = 0.432, *p*<0.05), and not for match trials (n = 28, *Spearman’s rho* = −0.088, *p*>0.05). This pattern is probably due to ceiling levels of performance for match trials in the photo-to-person task.

## Discussion

Consistent with previous research, our results emphasise that unfamiliar face matching is a difficult and error-prone task. Further, we show that this is not merely a laboratory phenomenon that is limited to novice participants. Trained passport officers also perform poorly when matching unfamiliar faces. High error rates were consistent across three tests, each of which was designed to emulate face matching in occupational settings. Further, across all experiments, length of time employed as a passport officer did not predict accuracy. Given the many face matching decisions made by passport officers as part of their daily workflow, we interpret this as strong evidence that experience alone does not improve accuracy on face matching tasks. To account for this result, we emphasise that although very experienced in face matching, passport officers rarely receive feedback on the accuracy of matching decisions. It is possible that they are not aware that unfamiliar face matching is a difficult task (a misconception that may stem from the ease with which people recognise *familiar* faces) and so are unlikely to learn from experience [9], [12].

Given the high error rates on this security-critical task, one might ask if there is anything that can be done to improve the situation. One possibility might be to provide more effective training. Our results suggest that current training methods in this workplace were not effective in improving matching accuracy. This disappointing finding is consistent with a previous evaluation of training courses that emphasised featural comparison of faces [10], [11]. However, alternative approaches to training based on performance feedback do appear to have promise, although the associated performance enhancements are modest [12].

An alternative solution would be to select staff on the basis of face matching aptitude. Our data suggest that this approach would confer an immediate and sizeable benefit to security. Across all experiments, we found large individual differences on face matching tests, with some people performing with 100% accuracy, and a significant proportion performing quite poorly (below 70% accuracy, on tasks where chance performance is 50%). This finding is consistent with a number of recent studies showing that performance in unfamiliar face matching tasks is subject to large individual differences [2], [3], [14]. Importantly, these individual differences appear to be highly stable across repeated testing on the same task [3], [15], suggesting that recruitment of high performers would be an effective strategy.

In parallel to this research, studies of face *memory*, as distinct from perceptual matching, show that some people are especially good at recognising familiar faces [14], while others have specific difficulties (for a review see [16]). Individual differences are almost certainly modulated by hereditary factors, as performance of monozygotic twins is strongly correlated [17]. Interestingly however, performance on face *memory* tasks only weakly predicts face *matching* ability [3], suggesting that these two modes of face identification rely on rather different cognitive processes. In future research it will be important to map in greater detail the degree of generalisation across different identification tasks. Understanding this profile will be critical in designing selection procedures for different occupations.

We propose that poor performance in face matching professionals is not confined to the particular workplace where we carried out this research, but is common to a wide range of occupational settings in which staff make face matching decisions as part of their daily work. Given this apparent vulnerability, recruitment testing for such roles should include aptitude tests that predict task performance; and these tests should be designed to emulate occupational task demands.

## Supporting Information

Performance Data S1 **Performance data for individual study participants.**
(XLSX)
